# Long noncoding RNA LINC00963 induces NOP2 expression by sponging tumor suppressor miR-542-3p to promote metastasis in prostate cancer

**DOI:** 10.18632/aging.103236

**Published:** 2020-06-17

**Authors:** Feng Sun, Ke Wu, Zhixian Yao, Xingyu Mu, Zhong Zheng, Menghao Sun, Yong Wang, Zhihong Liu, Yiyong Zhu

**Affiliations:** 1 Department of Urology, Shanghai General Hospital, Shanghai Jiao Tong University School of Medicine, Shanghai 200080, China

**Keywords:** lncRNA LINC00963, miR-542-3p, NOP2, metastasis, prostate cancer

## Abstract

Metastatic disease caused by castration-resistant prostate cancer (CRPC) is the principal cause of prostate cancer (PCa)-related mortality. CRPC occurs within 2–3 years of initiation of androgen deprivation therapy (ADT), which is an important factor of influencing PCa metastasis. Recent studies have revealed that non-coding RNAs in PCa can enhance metastasis and progression, while the mechanisms are still unclear. In this study, we reported that the long noncoding RNA-LINC00963 was increased in CRPC tissues and promoted migration of PCa cells in vitro and their metastasis in vivo. High levels of LINC00963 significantly decreased tumor suppressor miR-542-3p, whose levels in metastasis tissues were low compared to those in non-metastasis tissues. LINC00963 promotes and miR-542-3p inhibits metastasis. Furthermore, the expression levels of LINC00963 and miR-542-3p were positively and negatively associated with the expression of NOP2. We demonstrated that NOP2 promoted PCa by activating the epithelial-mesenchymal transition (EMT) pathway. For specific mechanism, dual luciferase reporter assays showed that miR-542-3p directly binds to both 3'-untranslated region (UTR) of LINC00963 and NOP2 mRNA. Taken together, our results show that LINC00963 acts as an inducer of PCa metastasis by binding miR-542-3p, thereby promoting NOP2. This axis may have diagnostic and therapeutic potential for advanced PCa.

## INTRODUCTION

Prostate cancer (PCa) remains the second major cause of male cancer-associated death in the United States. Androgen-deprivation therapy (ADT) is the primary treatment for patients with biochemically relapsed and metastatic PCa [1, 2]. Unfortunately, research has consistently shown that after an initial sensitivity to ADT, most patients generate resistance and thus progress to castration-resistant prostate cancer (CRPC) [3, 4]. Novel therapeutic drugs, namely cabazitaxel, abiraterone, and enzalutamide, have prolonged the survival of patients with CRPC [5–7]. However, outcomes for CRPC patients remain unsatisfying. CRPC is an essential cause of distant metastasis of prostate cancer, but the mechanism is still unclear [8, 9]. Hence, understanding the intrinsic mechanism of PCa metastasis is vitally crucial for future clinical management.

It has been observed for decades that non-protein-coding RNAs may possess significant cellular functions [10, 11]. The rapid advancement of high-resolution sequencing technologies allowed the detection of thousands of novel transcripts, now identified as long noncoding RNAs (lncRNAs), which participate in a broad spectrum of biological pathways, from gene transcription to protein translation [12, 13]. Recent research has established that aberrant regulation of lncRNAs plays pivotal roles in cell proliferation, chemo-resistance, and cancer metastasis in various malignancies [14, 15]. Further, lncRNAs can also function as competing endogenous RNAs (ceRNAs) by competitively binding to miRNAs and then modulating the targets of miRNAs, resulting in abnormal expression of downstream genes [11, 16, 17]. In terms of CRPC, several studies have indicated that lncRNA HOXD-AS1 (also known as HAGLR), PCAT1, and HORAS5 promote tumor proliferation and castration resistance and may serve as potential therapeutic targets [18–20]. In the same vein, ceRNA feedback loops like HOTAIR/EZH2/miR-193a, lncRNA CCAT1/DDX5/ miR-28-5p have shown a substantial effect on CRPC progression [21, 22]. Contrary to previous views, Chen and Gu have suggested that lncRNA LBCS can actively suppress the viability of CRPC by repressing Androgen Receptor (AR) signaling [23]. Although the function of lncRNAs in PCa has been widely investigated, the role of lncRNA in the process of tumorigenesis and downstream regulation of metastasis is still poorly characterized. Therefore, it is of considerable significance to illustrate the function of lncRNAs and their potential for use as diagnostic and therapeutic targets in PCa metastasis.

## RESULTS

### LINC00963 is increased in CRPC tissues

**Figure 1:**
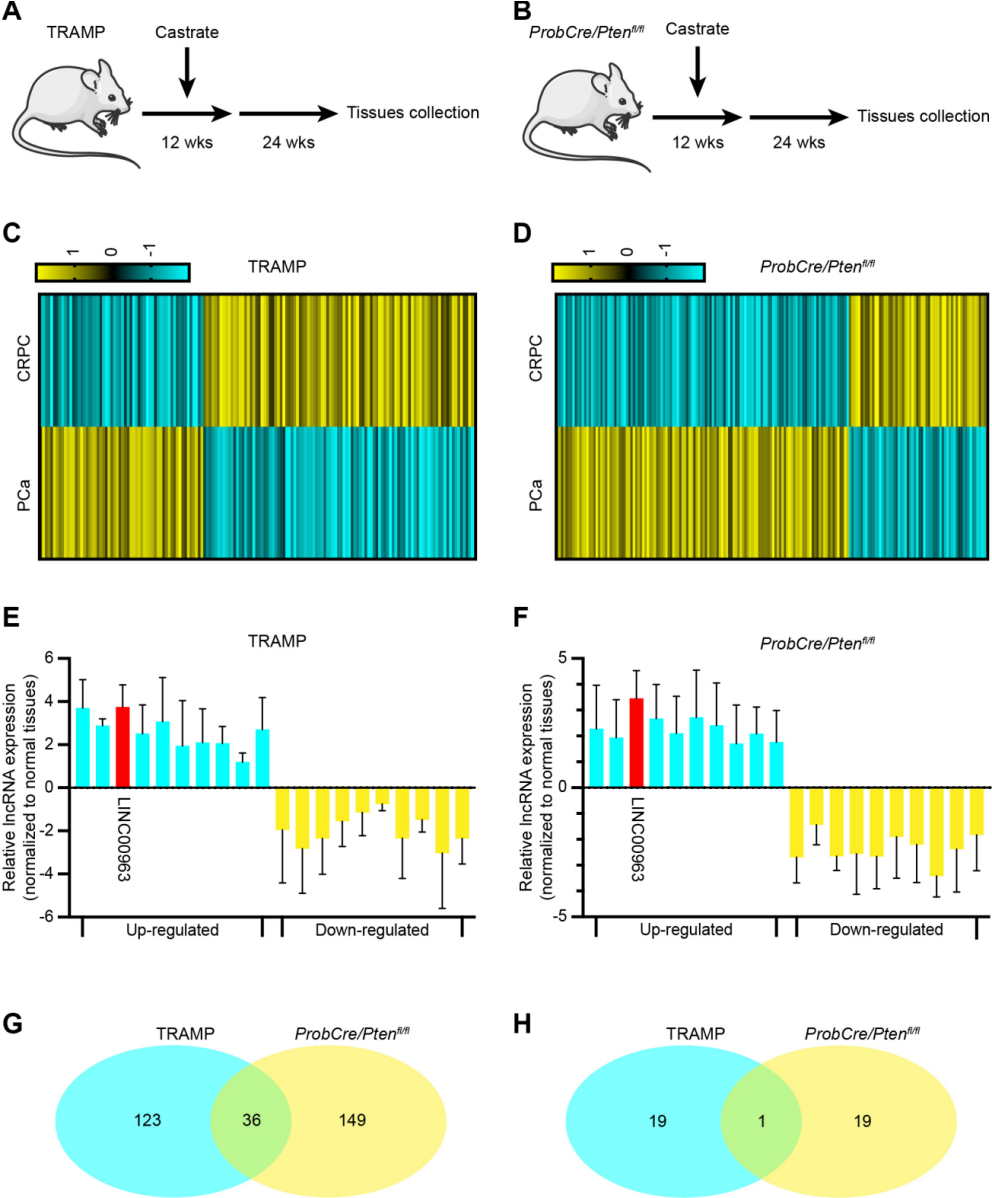
**Long noncoding RNA LINC00963 is increased in castration-resistant prostate cancer (CRPC) tissues.** (**A**, **B**) Experimental approach to construct CRPC mouse models; TRAMP mouse (**A**) and ProbCre/Pten^fl/fl^ mouse (**B**). (**C**, **D**) Heat map of differential expression of long noncoding RNAs (lncRNAs) in cells isolated from normal prostate cancer (PCa) and CRPC mouse models (**C**, TRAMP mouse; **D**, ProbCre/Pten^fl/fl^ mouse). (**E**, **F**) The expression of the 10 most apparent up- and decreased lncRNAs were analyzed by RT-qPCR in CRPC tissues and compared to those in normal PCa tissues (**E**, TRAMP mouse; **F**, ProbCre/Pten^fl/fl^ mouse). (**G**) Intersection of differentially expressed lncRNAs according to sequencing assays. (**H**) Intersection of differentially expressed lncRNAs according to RT-qPCR. Mean ± SEM.

After the transition to CRPC, PCa is prone to distant metastases. Thus, we explored the changes of lncRNAs between PCa and CRPC. As a CRPC mouse model, we used the TRAMP mouse model, in which the oncogene SV40 Tag is expressed under the control of the probasin promoter. As previously reported, TRAMP mice castrated at the age of 12 weeks gradually developed CRPC by the age of about 24 weeks (Figure 1A). These phenomena were also verified in the ProbCre/Ptenfl/fl prostate cancer model (Figure 1B). To identify lncRNAs that may be involved in the emergence of CRPC, we first analyzed RNA sequencing (RNA-seq) data of 3 CRPC tissues and 3 normal PCa tissues from the TRAMP and ProbCre/Pten^fl/fl^ mouse models. We found that the expression levels of 159 and 185 lncRNAs in CRPC tissues from the TRAMP mouse (Figure 1C) and the ProbCre/Pten^fl/fl^ mouse (Figure 1D) were significantly changed (Fold change > 2, P < 0.05) compared to those of normal PCa tissues. The expression levels of the top 10 increased and RT-qPCR examined decreased lncRNAs. The trends in the expression of lncRNAs in CRPC tissues from TRAMP mice (Figure 1E) and ProbCre/Pten^fl/fl^ mice (Figure 1F) were in line with the RNA-seq data. To explore the crucial lncRNA, we took the two sets of differentially expressed lncRNAs together. The results showed that there are 36 repeated lncRNAs in RNA-seq data (Figure 1G) and singular repeated lncRNA in RT-qPCR assays (Figure 1H).

### LINC00963 silencing inhibits CRPC cell metastasis

**Figure 2:**
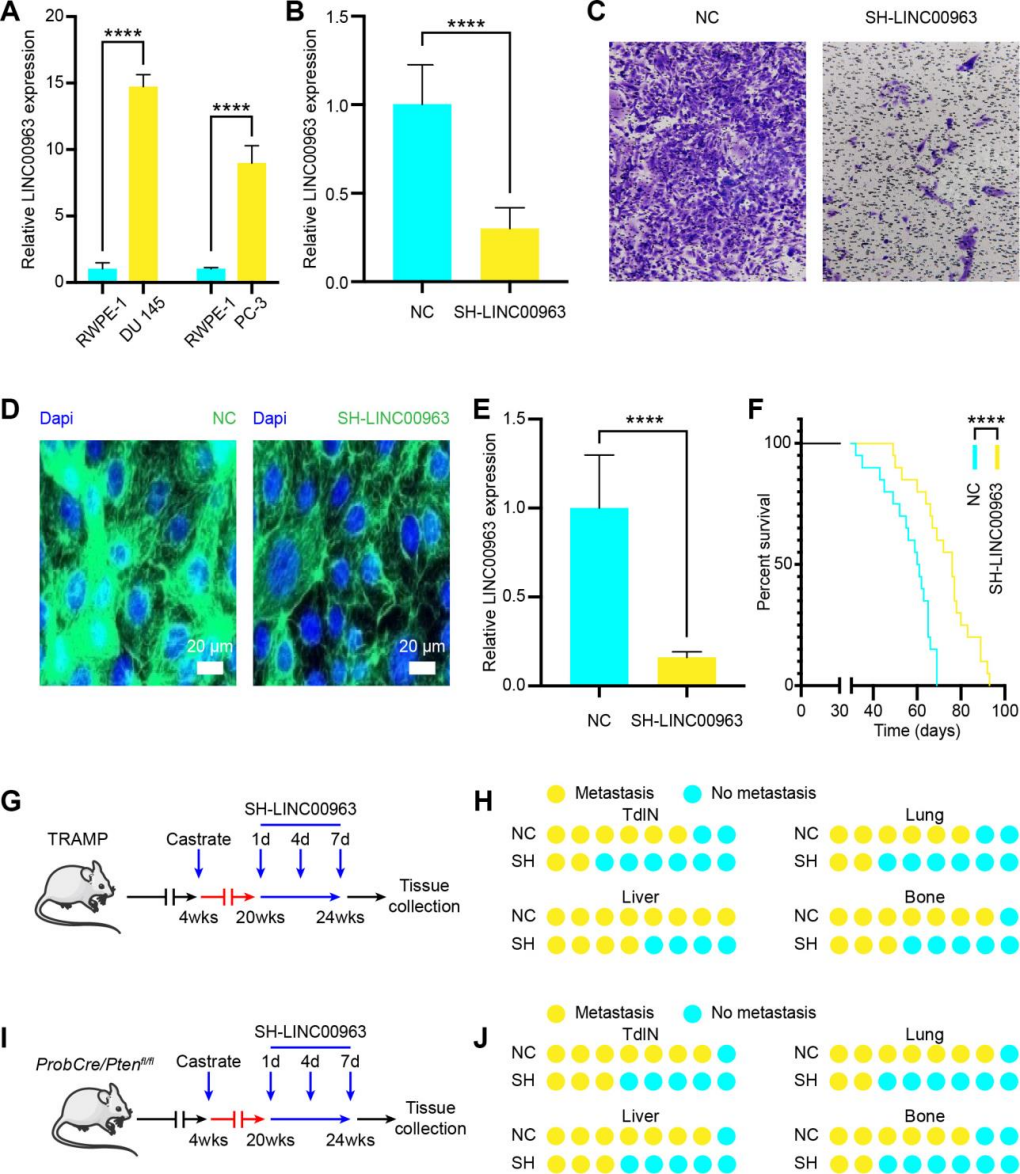
**Effects of noncoding RNA LINC00963 on PCa cell metastasis in vitro and in vivo.** (**A**) RT-qPCR analysis of LINC00963 expression in the PCa cell lines DU 145 and PC-3 compared to that of the normal prostate cell line RWPE-1. (**B**) LINC00963 expression in SH-LINC00963 Lentivirus and empty lentivirus vector–treated DU145 cells was tested using RT-qPCR. (**C**) Transwell invasion assays were performed to determine the invasion of SH-LINC00963 Lentivirus–transfected or empty lentivirus vector–transfected DU 145 cells. (**D**) A representative of the fluorescence image of LINC00963-GFP expression (green) and DAPI (blue) in SH-LINC00963 lentivirus–infected prostate of PCa mice. Scale bars = 20 μm. (**E**) RT-qPCR analysis of LINC00963 expression in SH-LINC00963 Lentivirus–transfected or empty lentivirus vector–transfected PCa tissues. (**F**) Survival time of PCa mice injected with SH-LINC00963 Lentivirus or empty lentivirus vector. (**G**) Experimental approach to knock down LINC00963 in castrated TRAMP mice. (**H**) Incidence of TdLN, liver, lung, and bone metastases following injection with SH-LINC00963 Lentivirus or empty lentivirus vector in castrated TRAMP mice (n = 10, every group). (**I**) The experimental approach to knock down LINC00963 in castrated ProbCre/Pten^fl/fl^ mice. (**J**) Incidence of TdLN, liver, lung, and bone metastases following injection with SH-LINC00963 Lentivirus or empty lentivirus vector in castrated ProbCre/Pten^fl/fl^ mice (n = 10, every group). Mean ± SEM, ****P < 0.001.

To investigate the functions of LINC00963 in PCa cells metastasis, we examined LINC00963 expression levels in the PCa cell lines DU 145 (PCa cells extracted from brain metastases) and PC-3 (PCa cells extracted from bone metastases). We compared them to those in the normal prostate cell line, RWPE-1. The RT-qPCR analysis showed that the levels of LINC00963 in DU 145 and PC-3 cells were significantly higher than those in RWPE-1 cells (Figure 2A). Then, we knocked down LINC00963 in DU 145 cells by transfection with the SH-LINC00963 lentivirus. The expression of LINC00963 was effectively decreased in cells transfected with the SH-LINC00963 lentivirus compared with that in cells transfected with the empty lentivirus vector (Figure 2B). Furthermore, transwell invasion assays showed that LINC00963 knockdown significantly inhibited DU 145 cell invasion (Figure 2C). AAV lentiviral can be used as a vector to regulate lncRNAs levels in vivo [24]. To examine the in vivo function of LINC00963, we generated the lentiviral vector contained GFP to down-regulate the expression of LINC01234 in the prostate gland. Following injection to the tail vein, SH-LINC00963 lentivirus effectively infected the prostate, produced considerable LINC00963-GFP signal (Figure 2D), and significantly decreased the amount of LINC00963 in vivo (Figure 2E). Low levels of LINC00963 also resulted in significantly prolonged survival time (Figure 2F). To investigate the role of LINC00963 in the onset of CRPC and PCa metastasis, we used SH-LINC00963 lentivirus to knock down LINC00963 during carcinogenesis of castrated TRAMP mice (Figure 2G). We injected SH-LINC00963 lentivirus every 3 days at 8 weeks after castration for a total of 4 times. Histopathological analysis revealed that, compared to that of the isotype vector controls, the downregulation of LINC00963 resulted in significantly reduced incidence of tumor draining lymph nodes (TdLNs), liver, lung, and bone metastases (Figure 2H). To verify these results, we used SH-LINC00963 lentivirus to knock down LINC00963 during carcinogenesis of castrated ProbCre/Pten^fl/fl^ mice and obtained similar results (Figure 2I). These results were also verified in the ProbCre/Pten^fl/fl^ prostate cancer mouse model (Figure 2J). Taken together, these results showed that silencing LINC00963 could effectively inhibit progression of PCa-CRPC-metastasis.

### LINC00963 functions as a ceRNA and sponges miR-542-3p

**Figure 3:**
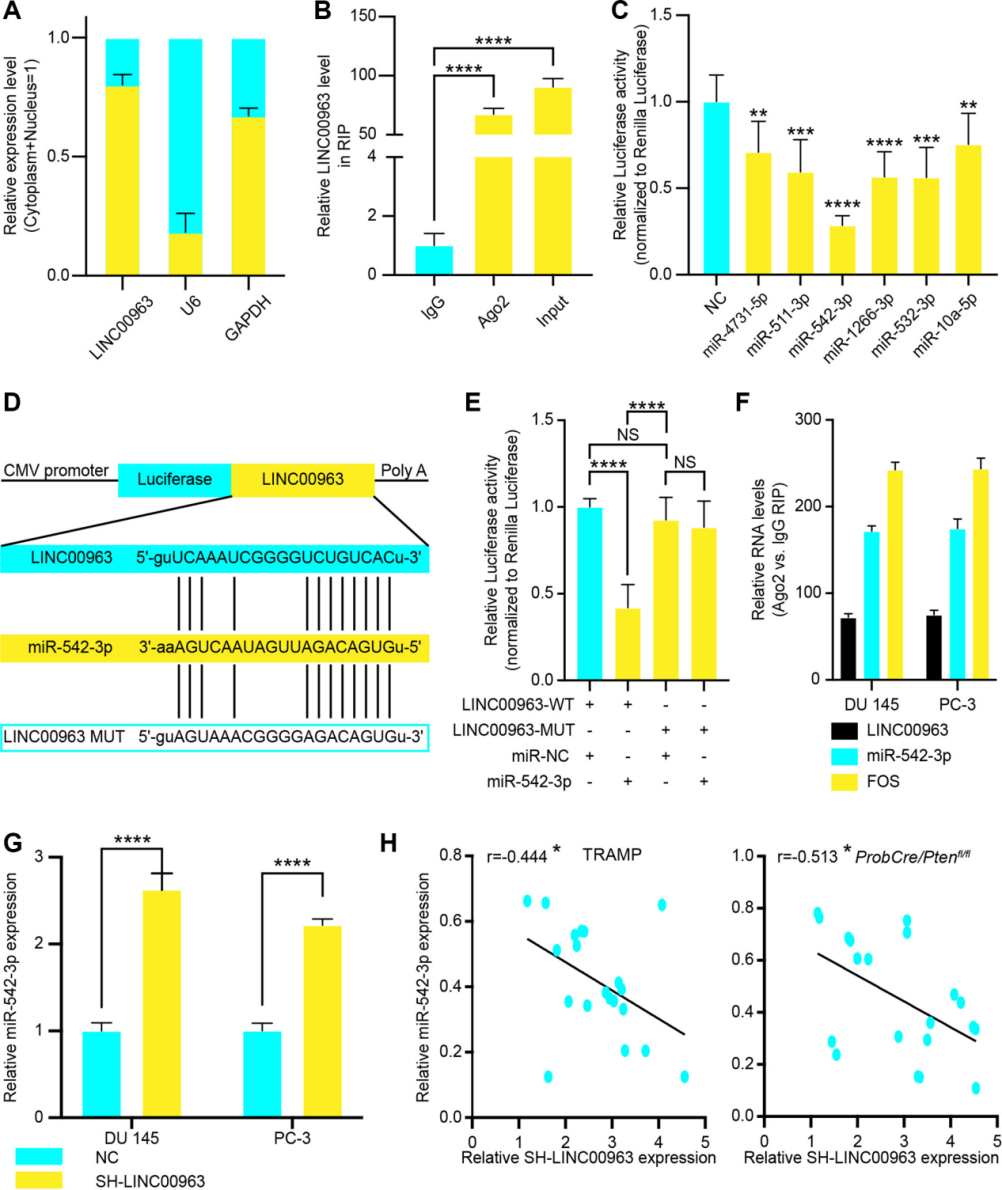
**The relationship between long noncoding RNA LINC00963 and tumor suppressor miR-542-3p.** (**A**) The quality of LINC00963 in DU 145 cytoplasmic and nuclear fractions. Levels of glyceraldehyde-3-phosphate dehydrogenase (GAPDH) mRNA and U6 snRNA in purified nuclear fractions were detected by reverse transcription quantitative polymerase chain reaction (RT-qPCR). (**B**) RIP experiments were performed in DU 145 cells, and the coprecipitated RNA was subjected to RT-qPCR for LINC00963. The fold enrichment of LINC00963 in argonaute 2 (Ago2) RIP is higher relative to that of its matching immunoglobulin (IgG) control. (**C**) The luciferase reporter plasmid (RLuc-LINC00963) was co-transfected into 293T cells with the 6 miRNA-coding plasmids. (**D**, **E**) The luciferase reporter plasmid containing wild-type (WT) or mutant (Mut) LINC00963 (**D**) was co-transfected into 293T cells with miR-542-3p or with an empty plasmid vector (**E**). (**F**) RNA levels in Ago2 immunoprecipitates are presented as fold enrichment relative to IgG immunoprecipitates. (**G**) RT-qPCR analysis of miR-542-3p expression in DU 145 and PC3 cells transfected with empty lentivirus vector or SH-LINC00963 Lentivirus. (**H**, **I**) Correlation of LINC00963 and miR-542-3p in CRPC tissues from TRAMP (**H**) and ProbCre/Pten^fl/fl^ mice (**I**). Mean ± SEM, *P<0.05, **P < 0.01, ***P < 0.005, ****P < 0.001.

Recent studies have shown that lncRNAs can regulate target gene expression by interacting with RNA-binding proteins, such as polycomb repressive complex 2 (PRC2), or by acting as ceRNAs for miRNAs [25]. To investigate the molecular mechanism by which LINC00963 promotes PC cell metastasis, we first analyzed its subcellular localization. RT-qPCR assayed the levels of LINC00963 in nuclear and cytoplasmic fractions. The isolated nuclear fraction displayed high levels of nuclear markers (U6 snRNA) but low levels of cytoplasmic markers (GAPDH). Furthermore, LINC00963 was found to be more abundant in the cytoplasm than in the nucleus (Figure 3A), suggesting that LINC00963 may regulate target gene expression at the posttranscriptional level. Indeed, RNA binding protein immunoprecipitation assays using PCa cell extracts revealed that LINC00963 binds directly to Ago2, a component of the RNA-induced silencing complex involved in miRNA-mediated repression of mRNAs (Figure 3B). This finding suggested that LINC00963 may function as a ceRNA of miRNAs. To examine this hypothesis, we used online bioinformatics databases (DIANA Tools: http://diana.imis.athena-innovation.gr and Starbase: http://starbase.sysu.edu.cn/) and observed that LINC00963 sequence contains potential miR-4731-5p, miR-511-3p, miR-542-3p, miR-1266-3p, miR-532-3p, and miR-10a-5p binding sites. We then performed dual luciferase reporter assays to confirm the prediction analysis. 293T cells were transfected with a luciferase plasmid harboring the sequence of LINC00963 together with plasmids encoding the miRNAs mentioned above or a control sequence. We found that miR-4731-5p, miR-511-3p, miR-542-3p, miR-1266-3p, miR-532-3p, and miR-10a-5p could suppress LINC009634-driven luciferase activity, and the suppression ability of miR-542-3p was stronger (Figure 3C). Hence, we chose miR-542-3p for further investigation and constructed a reporter construct in which the putative miR-542-3p binding site in the LINC009634 sequence was mutated by site-directed mutagenesis (Figure 3D). As expected, the miR-542-3p–mediated suppression of luciferase activity was abolished by the mutant LINC009634 (Figure 3E). In addition, RNA-binding protein immunoprecipitation experiments showed that LINC009634 and miR-542-3p were enriched in Ago2 immunoprecipitates compared to those of the control IgG (Figure 3F). We next evaluated the levels of miR-542-3p in DU 145 and PC-3 cells after knocking down the expression of LINC009634. Notably, the LINC009634 knockdown significantly increased the expression levels of miR-542-3p (Figure 3G). Furthermore, we collected CRPC tissues form TRAMP and ProbCre/Pten^fl/fl^ mice and tested LINC009634/miR-542-3p levels by RT-qPCR. The results showed that there is a negative relationship between LINC009634 and miR-542-3p (Figure 3H and 3I). Taken together, these results showed that LINC00963 played as a sponge to decreased miR-542-3p.

### MiR-542-3p overexpression inhibits PCa cell metastasis

**Figure 4:**
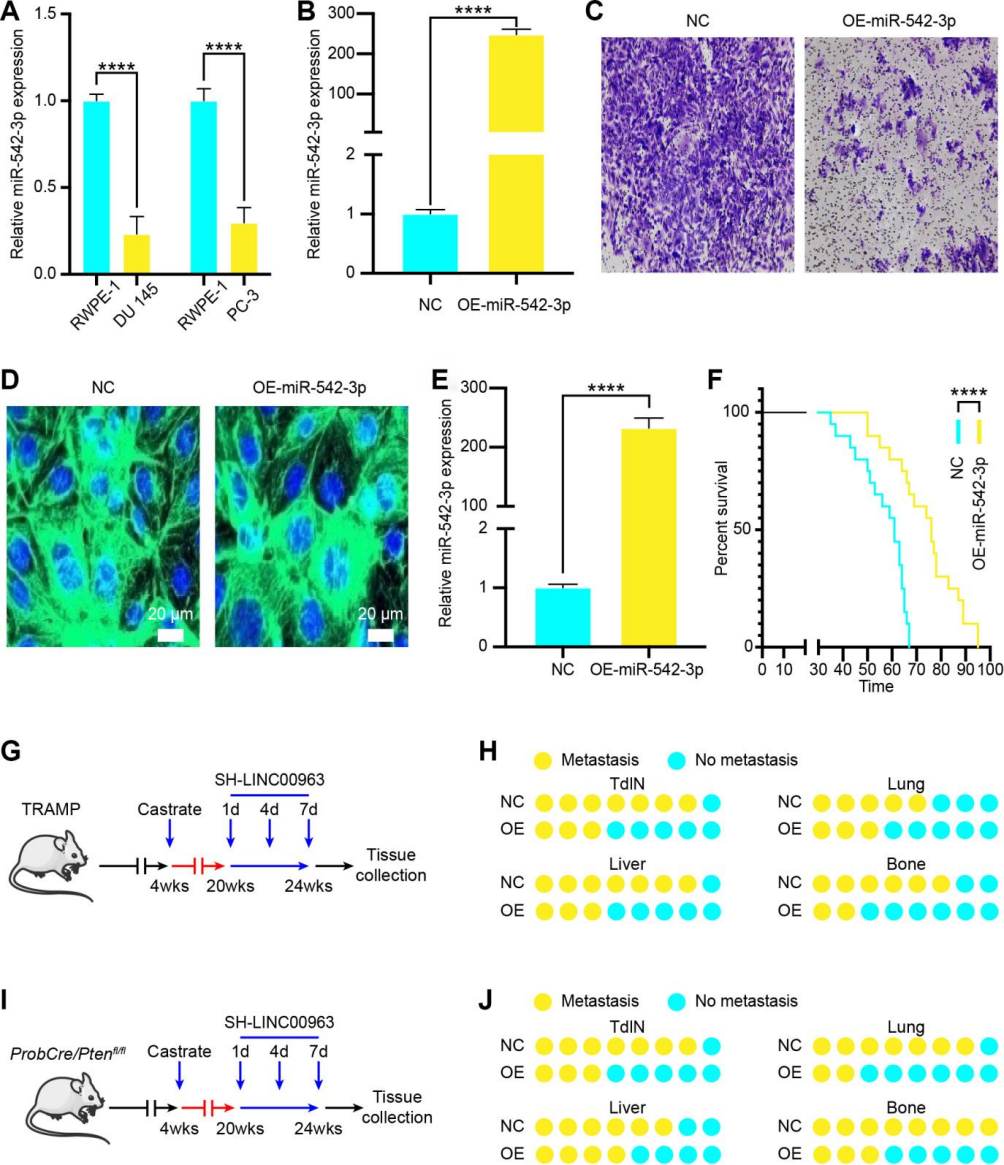
**Effects of tumor suppressor miR-542-3p on PCa cell metastasis in vitro and in vivo.** (**A**) RT-qPCR analysis of miR-542-3p expression in the PCa cell lines DU 145 and PC-3 compared to that of the normal prostate cell line RWPE-1. (**B**) MiR-542-3p expression in OE-miR-542-3p Lentivirus- and empty lentivirus vector-transfected DU145 was tested by RT-qPCR. (**C**) Transwell invasion assays were performed to determine the invasion of OE-miR-542-3p Lentivirus– or empty lentivirus vector–transfected DU 145 cells. (**D**) A representative fluorescence image of miR-542-3p expression (green) and DAPI (blue) in OE-miR-542-3p-GFP- or empty lentivirus vector-infected prostate of PCa mice. Scale bars = 20 μm. (**E**) RT-qPCR analysis of miR-542-3p expression in OE-miR-542-3p Lentivirus– or empty lentivirus vector–transfected PCa tissues. (**F**) Survival time of PCa mice with OE-miR-542-3p Lentivirus– or empty lentivirus vector–transfected. (**G**) Experimental approach to knock down miR-542-3p in castrated TRAMP mice. (**H**) Incidence of TdLNs, liver, lung, and bone metastases following OE-miR-542-3p Lentivirus–transfection or pcDNA-miR-542-3p lentivirus–transfection of castrated TRAMP mice (n = 10, every group). (**I**) Experimental approach to knock down miR-542-3p in castrated ProbCre/Pten^fl/fl^ mice. (**J**) Incidence of TdLNs, liver, lung, and bone metastases following OE-miR-542-3p Lentivirus–transfection or pcDNA-miR-542-3p lentivirus–transfection in castrated ProbCre/Pten^fl/fl^ mice (n = 10, every group). Mean ± SEM, ****P < 0.001.

To investigate the function of miR-542-3p in PCa cells, we examined miR-542-3p expression levels in the PCa cell lines DU 145 and PC-3 and compared them to the normal prostate cell line RWPE-1. The RT-qPCR analysis showed that the expression of miR-542-3p in DU 145 and PC-3 cells was significantly lower than that in RWPE-1 cells (Figure 4A). Then, we transfected DU 145 with OE-miR-542-3p lentivirus or with empty lentivirus vector and found that OE-miR-542-3p lentivirus effectively increased miR-542-3p (Figure 4B). Furthermore, transwell invasion assays showed that miR-542-3p overexpression significantly inhibited DU 145 cell invasion (Figure 4C). To examine the in vivo effects of miR-542-3p overexpression, we generated the lentiviral vector LV-miR-542-3p-GFP to overexpress miR-542-3p. Tail vein injection of LV-miR-542-3p-GFP effectively infected the prostate, produced considerable miR-542-3p-GFP signal (Figure 4D), and significantly increased the amount of miR-542-3p in vivo (Figure 4E). Furthermore, injected mice showed a significantly prolonged survival time (Figure 4F). To investigate the role of miR-542-3p in the onset of CRPC, we used LV-miR-542-3p-GFP to knock down miR-542-3p during carcinogenesis of castrated TRAMP mice (Figure 4G). We injected LV-miR-542-3p-GFP every 3 days at 8 weeks after castration for a total of four times. Histopathological analysis revealed that, compared to the isotype vector controls, overexpression of miR-542-3p resulted in significantly reduced incidence of TdLNs, liver, lung, and bone metastases in these mice (Figure 4H). To verify these results, we used LV-miR-542-3p-GFP to overexpress miR-542-3p during carcinogenesis of castrated ProbCre/Ptenfl/fl mice with similar results (Figure 4I). These results were also verified in the ProbCre/Ptenfl/fl prostate cancer mouse model (Figure 4J). Taken together, these results showed that overexpression miR-542-3p could effectively inhibit the metastasis of PCa.

### NOP2 is a miR-542-3p target gene

**Figure 5:**
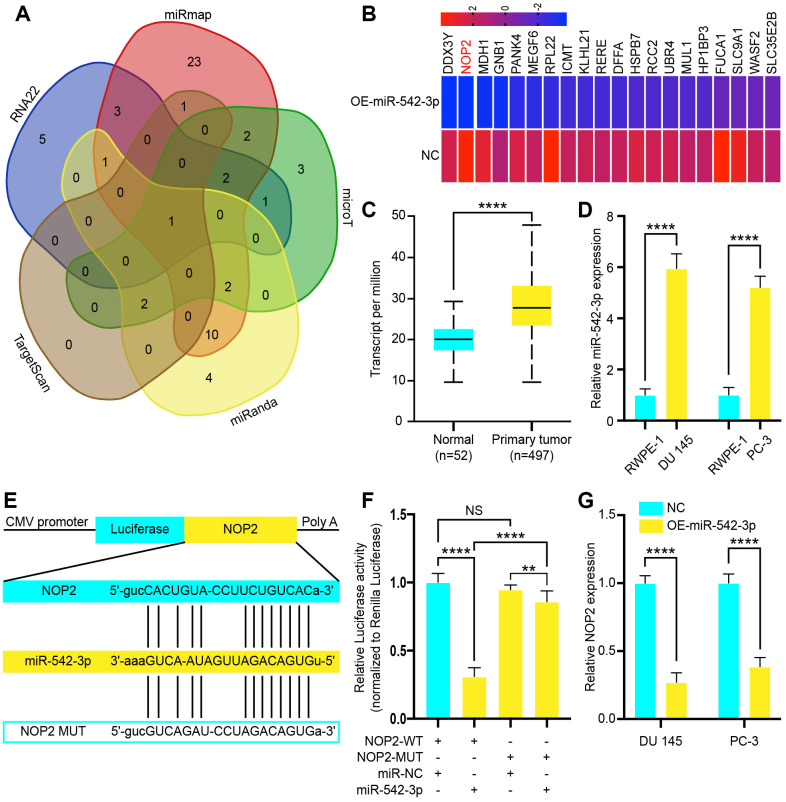
**NOP2 is a target of tumor suppressor miR-542-3p and is suppressed by long noncoding RNA LINC00963 deletion.** (**A**) Venn diagram of intersection target genes of miR-542-3p predicted by several bioinformatics databases. (**B**) Heat map of top 20 decreased genes in DU 145 cells after transfection with OE-miR-542-3p Lentivirus. (**C**) Relative expression of NOP2 in PCa tissues compared to that of normal tissue was analyzed by using the TCGA dataset. (**D**) RT-qPCR analysis of NOP2 expression in PCa cell lines the DU 145 and PC-3 cells compared to the normal prostate cell line RWPE-1. (**E**) Schematic view of miR-542-3p putative targeting site in the WT and Mut 3’-untranslated region (UTR) of *NOP2*. (**F**) Luciferase activity assay in 293 T cells transfected with luciferase report plasmids containing NOP2 3’UTR (WT or Mut), and control miRNA or miR-542-3p. (**G**) Relative mRNA levels of NOP2 in DU 145 and PC-3 cells transfected with control vector or OE-miR-542-3p Lentivirus. Mean ± SEM, **P < 0.01, ****P < 0.001, NS: no statistical significance.

To determine the target gene of miR-542-3p in PCa, we used RNA22 (http://cm.jefferson.edu/rna22v1.0/), miRmap (http://mirmap.ezlab.org/), microT (http://www.microrna.gr/microT), miRanda (http://www.microrna.org/microrna/home.do) and Target Scan (http://www.targetscan.org/) to predict potential miR-542-3p target genes (Figure 5A). This analysis showed that NOP2 was the only gene at the intersection of all databases, which is also a potential target gene of LINC00963. Next, these observations were echoed by RNA-seq, which revealed that the expression of NOP2 was reduced in DU 145 transfected with OE-miR-542-3p Lentivirus (Figure 5B). Then, we analyzed The Cancer Genome Atlas (TCGA) RNA-seq data and found that NOP2 was increased in PCa tissues (Figure 5C). Therefore, we chose NOP2 for further investigation and examined its expression levels in the PCa cell lines DU 145 and PC-3 and compared them to those in the normal prostate cell line RWPE-1. The RT-qPCR analysis showed that expression of NOP2 in DU 145 and PC-3 was significantly higher than that in RWPE-1 cells (Figure 5D). Then, we constructed a reporter construct in which the putative miR-542-3p binding site in the NOP2 sequence was mutated by site-directed mutagenesis (Figure 5E). As expected, the miR-542-3p–mediated suppression of luciferase activity was abolished by the mutant NOP2 (Figure 5F). We next evaluated the expression level of NOP2 in DU 145 and PC-3 cells after overexpression of miR-542-3p. Notably, miR-542-3p overexpression significantly decreased the expression levels of NOP2 (Figure 5G). Collectively, these data suggest that miR-542-3p decreases the expression levels of NOP2.

### NOP2 is a miR-542-3p target gene and is indirectly regulated by LINC00963

**Figure 6:**
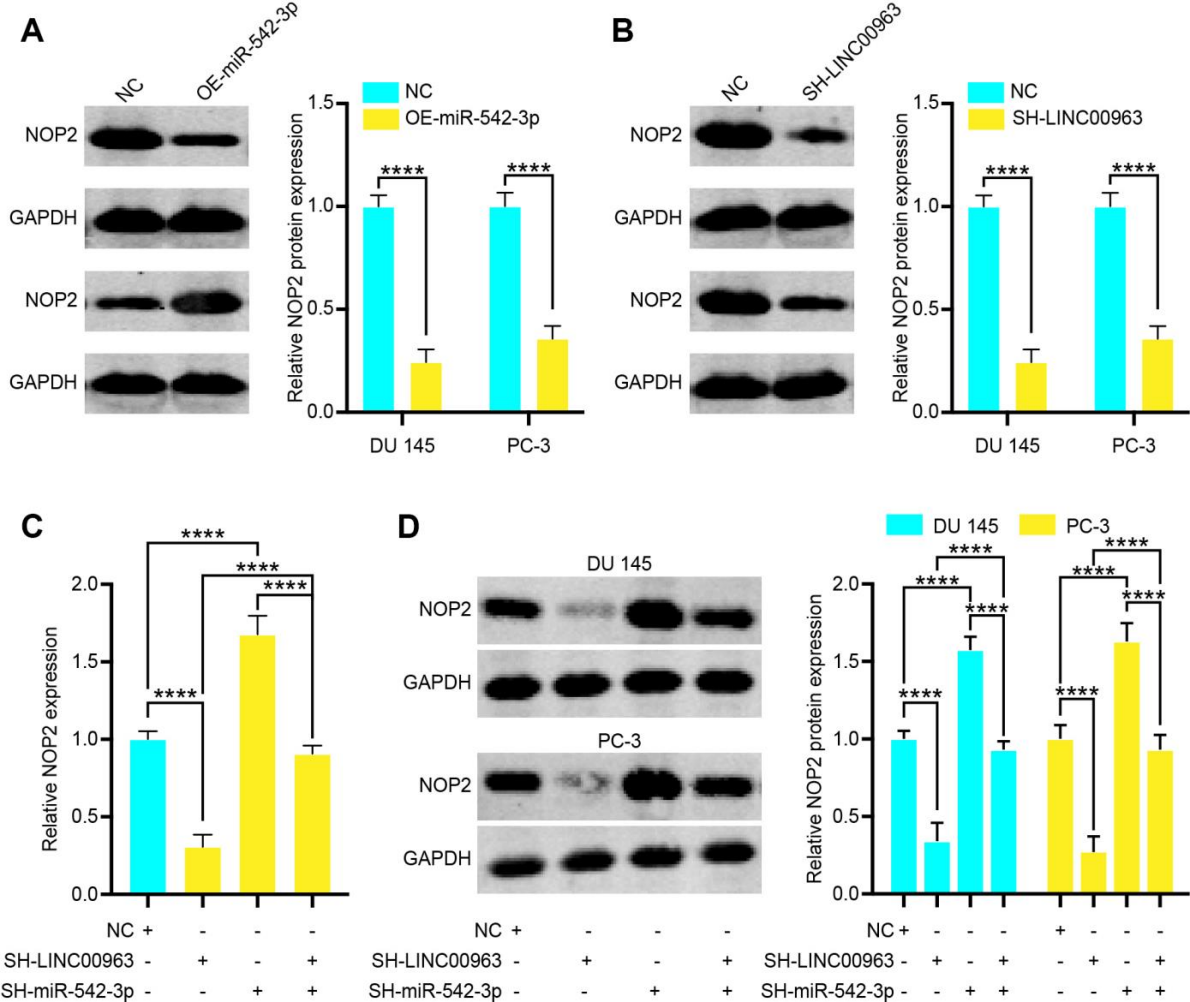
**Long noncoding RNA LINC00963 functions as a competing endogenous RNA (ceRNA) and sponges tumor suppressor miR-542-3p to upregulate NOP2.** (**A**) Relative protein levels of NOP2 in DU 145 cells transfected with empty vector lentivirus or OE-miR-542-3p lentivirus. (**B**) Relative protein levels of NOP2 in DU 145 cells transfected with empty vector lentivirus or SH-LINC00963 Lentivirus. (**C**, **D**) NOP2 mRNA (**C**) in 293T cells and protein level in DU 145 cells and PC-3 cells (**D**) following knockdown of LINC00963 and/or inhibition of miR-542-3p. Mean ± SEM, ****P < 0.001.

As LINC00963 can sponge miR-542-3p, we next examined whether LINC00963 can regulate the expression of NOP2 by binding to miR-542-3p. We found that overexpression of miR-542-3p increased protein levels of NOP2 in DU 145 cells (Figure 6A). Next, we knocked down LINC00963 and found that decreased LINC00963 also significantly reduced NOP2 protein levels in DU 145 cells (Figure 6B). To determine whether miR-542-3p plays a role in the relationship between LINC00963 and NOP2, we co-transfected cells with SH-LINC00963 Lentivirus and the SH-miR-542-3p Lentivirus. The suppression of NOP2 mRNA in 293T cells and protein levels in DU 145 and PC-3 cells by SH-LINC00963 Lentivirus was effectively reversed by the SH-miR-542-3p Lentivirus (Figure 6C and 6D). Collectively, these data suggest that LINC00963 modulates the expression of NOP2 by posttranscriptional regulation of miR-542-3p.

### NOP2 promotes PCa metastasis by epithelial-mesenchymal transition (EMT) pathway

**Figure 7:**
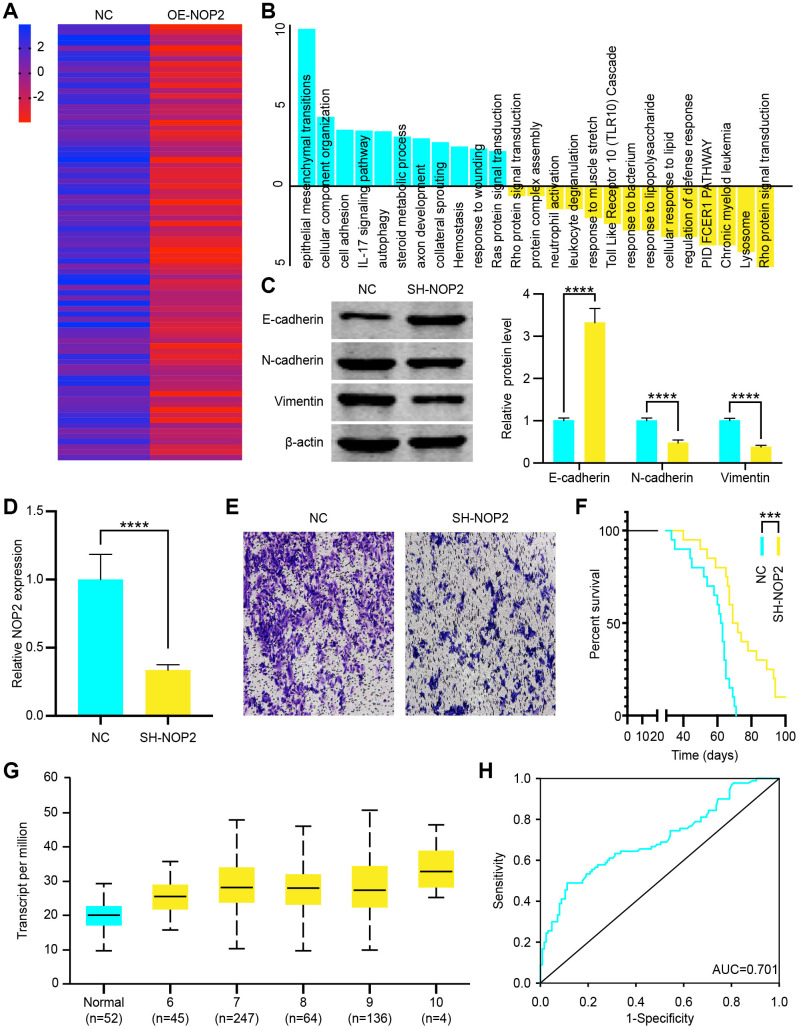
**NOP2 promotes prostate cancer (PCa) cell progression through epithelial-mesenchymal transition (EMT) pathway.** (**A**) Heat map of increased genes in DU 145 cells transfected with OE-NOP2 Lentivirus. (**B**) Gene enrichment analysis showed the signaling pathways activated or inhibited by NOP2 overexpression. (**C**) EMT related proteins (E-cadherin, N-cadherin, and vimentin) was examined by western blot in vector or OE-NOP2 Lentivirus transfected cells and the data were quantitated. (**D**) NOP2 expression in SH-NOP2 Lentivirus- and pcDNA-NOP2 Lentivirus–transfected DU145 cells was examined by RT-qPCR. (**E**) Transwell invasion assays were performed to determine the invasion of SH-NOP2 Lentivirus–or pcDNA-NOP2 Lentivirus–transfected DU 145 cells. (**F**) Survival time of PCa mice transfected with SH-NOP2 Lentivirus or pcDNA-NOP2 Lentivirus. (**G**) Transcript levels of NOP2 in normal prostate tissues and PCa tissues and differential Gleason scores. (**H**) Receiver operation characteristic (ROC) curve analysis of the association of NOP2 with the diagnosis of PCa (AUC = 0.701). Mean ± SEM, ***P < 0.005, ****P < 0.001.

To investigate the oncogenic role of NOP2 in PCa, we overexpressed it in DU 145 cells, and positively-regulated genes were analyzed. The heat map showed that there were 82 genes highly increased in OE-NOP2 Lentivirus transfected cells (fold change>2, P<0.05 = (Figure 7A). We further analyzed the signaling pathways of these increased genes and found that the EMT signaling pathway was significantly activated (Figure 7B). Similarly, DU 145 cells were transfected with OE-NOP2 Lentivirus, and the EMT signaling pathway positively related protein, E-cadherin, was increased and the negatively related proteins N-cadherin and Vimentin were decreased (Figure 7C). To verify cancer promoting function of NOP2, we transfected DU 145 cells with SH-NOP2 Lentivirus to knock down its expression, which was confirmed by RT-qPCR (Figure 7D). Then, the transwell invasion assays showed that decreased NOP2 significantly inhibited DU 145 cell invasion (Figure 7E). NOP2 knockdown also resulted in a significantly prolonged survival of mice (Figure 7F). To examine its clinical significance, we analyzed NOP2 expression in PCa and normal tissues. The expression of NOP2 was found to be increased in PCa samples compared with normal tissues according to TCGA sequencing data, and NOP2 levels were positively related to Gleason score (Figure 7G). Moreover, the ROC curve analysis showed that NOP2 could be regarded as a potential diagnosis indicator of PCa (Figure 7J). These findings indicate that NOP2 behaves as an oncogene to promote PCa cell proliferation, and can serve as a clinical indicator.

## DISCUSSION

Emerging data have shown that lncRNAs exert a crucial influence in human ailment and cellular function. Dysregulation of lncRNAs may also lead to tumorigenesis and cancer drug resistance [26, 27]. We discovered that LINC00963 is a novel CRPC–related lncRNA, which is significantly increased in PCa tissues and cell lines. Higher expression of LINC00963 correlated with advanced TNM stage, larger tumor size, and positive lymph node invasion. Moreover, elevated LINC00963 expression was associated with shorter overall survival (OS) and progression-free survival (PFS) time of patients with CRPC. Experiments in vitro and in vivo indicated that the inhibition of LINC00963 could restrain cell cycle and cell proliferation and enhance cell death, whereas overexpression of LINC00963 had the opposite effects. Collectively, these discoveries suggest the notion that LINC00963 possesses a critical role in PCa oncogenesis and could be considered as a novel treatment target in CRPC.

According to previous reports, there are novel and widespread interactive networks, including ceRNAs, where lncRNAs could interact with protein-coding mRNAs in a miRNA-dependent manner [28, 29]. For instance, lncRNA HOTAIR functions as a promoter of PCa cell proliferation by acting as a ceRNA for miR-193a [21]. lncRNA CCAT1 promotes CRPC invasion and facilitates the expression of AR-regulated genes by acting as a ceRNA of miR-28-5p [22]. In this study, we confirmed that LINC00963 localized in the cytoplasm and that it interacts with Ago2 in PCa cells, suggesting its potential role as an endogenous miRNA sponge. After searching various bioinformatical databases and applying luciferase reporter assays, we confirmed that miR-542-3p is a novel target of LINC00963. Regarded as a tumor suppressor, miR-542-3p presents low expression in various types of human cancers.

Lyu and colleagues have found that miR-542-3p inhibits breast cancer cell proliferation and HER3 signaling-induced chemo-resistance [30]. In addition, upregulating the expression of miR-542-3p could suppress the progression of colorectal cancer cells [31]. Furthermore, it has shown that miR-542-3p suppresses cell proliferation by inhibiting CDK14 in epithelial ovarian cancer [32]. In this study, we also found that miR-542-3p was significantly decreased in CRPC and increased miR-542-3p prevented PCa cell growth and increased the rate of apoptotic cell.

Generally, miRNA targets are an essential part of ceRNA networks where lncRNAs can exert their biological function. Using reliable online predicting tools, we revealed that NOP2 is one of the potential miR-542-3p targets. RNA-binding protein NOP2 can increase nucleolar activity and stimulate cell proliferation by influencing the cell cycle. In agreement with previous research reporting NOP2 in hepatocellular carcinoma [33], we found that NOP2 was highly expressed in CRPC tissues compared with the normal ones. PCa patients with higher levels of NOP2 demonstrate poorer OS. Furthermore, we showed that knockdown of NOP2 repressed PCa cell growth and induced cell death. Rescue experiments also confirmed that after knocking down NOP2, the inhibition effect induced by miR-542-3p down-regulation partially reversed.

**Figure 8:**
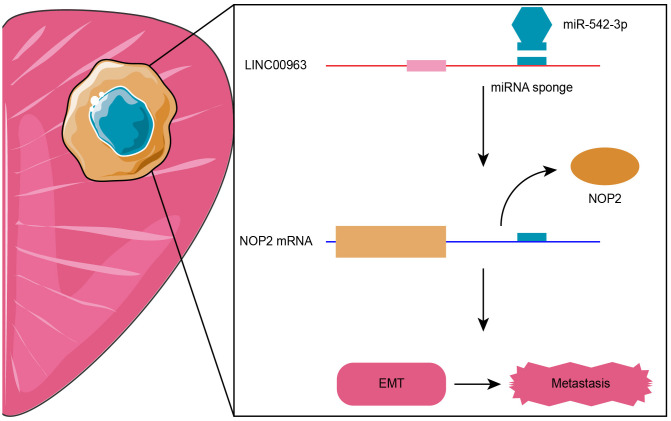
**Proposed model of LINC00963/miR-542-3p/NOP2 axis-mediated epithelial-mesenchymal transition (EMT) pathway activation for promoting metastasis in prostate cancer (PCa).**

In summary, lncRNA-LINC00963 functions as a ceRNA that competitively binds to miR-542-3p, which then up-regulates NOP2 and promotes PCa metastasis via activating EMT signaling, which is the critical pathway regulating tumor metastasis in several cancers [34, 35]. The LINC00963/miR-542-3p/NOP2 axis was shown to be involved in the development of CRPC, and targeting this pathway might have therapeutic potential for CRPC (Figure 8).

## MATERIALS AND METHODS

### Animals

All animal studies were approved by the Institutional Animal Care and Use Committee of Shanghai Jiao Tong University. All mice were maintained in pathogen-free conditions and cared for in compliance with the ethical regulations at Shanghai General Hospital. Transgenic adenocarcinomas of mouse prostate (TRAMP) and ProbCre/Pten^fl/fl^ mice were provided by the Shanghai Model Organisms Center (Shanghai, China) sacrificed 12 weeks post-castration unless indicated otherwise [36]. We tested androgen to determine model successfully established. Prostates, TdLNs, livers, lungs, and bones were collected for hematoxylin and eosin (H&E) and immunohistochemistry staining. The diagnosis of tumor metastasis was made independently by two pathologists. The genetic background of all mice used in this study was C57BL/6.

### Cell culture

Two human prostate cancer cell lines (DU 145, and PC-3) and a normal prostate cell line (RWPE-1) were purchased from the Institute of Biochemistry and Cell Biology of the Chinese Academy of Sciences (China). DU 145 and PC-3 cells were cultured in Ham’s F12 medium and RWPE-1 cells were cultured in K-SFM medium supplemented with 10% fetal bovine serum (FBS), 100 mg/mL streptomycin (Invitrogen), and 100 U/mL penicillin in humidified air at 37 °C with 5% CO_2_. All cell lines were authenticated by short tandem repeat DNA profiling.

### RNA extraction and quantitative RT-qPCR assays

Total RNA was extracted from tissues or cultured cells using TRIzol reagent (Invitrogen) according to the manufacturer's instructions. RNA (1 mg) was reverse transcribed in a final volume of 20 mL using random primers under standard conditions using the PrimeScript RT Reagent Kit (TaKaRa). Real-time PCR analyses were performed with SYBR Premix Ex Taq (TaKaRa). Levels of LINC00963 (primer: forward 5'-TGGACA CCACTTTGCCCTTT-3', reverse: 5'-AGATGGGGCC CTTATCACCT-3') and NOP2 (primer: forward 5'-AAATGGGAGAAGGTGGCGTC-3', reverse: 5'-CTCTCGGACATTAACCCGCA-3') were normalized to the expression of glyceraldehyde-3-phosphate dehydrogenase (GAPDH). Primers for miR-542-3p (primer: forward 5'-TCGGGGATCATCATGTCACG-3', reverse: 5'-GAGTGGCTCCCAGACCTTTC-3') and U6 (miRNA0002-1-200) were purchased from RiboBio. RT-qPCR and data collection were conducted on an ABI 7500 real-time PCR system (Applied Biosystems). RT-qPCR results were analyzed and expressed relative to the threshold cycle (Ct) values, and then converted to fold changes.

### Transduction of BC cell lines and RNA extraction

The short hairpin RNA (shRNA) and green fluorescent protein (GFP)-labeled lentivirus vectors containing the miR-542-3p mimic lentivirus (OE-miR-542-3p), LINC00963 overexpression lentivirus (OE-LINC00963), NOP2 overexpression lentivirus (OE-NOP2), miR-542-3p inhibitor lentivirus (SH-miR-542-3p), LINC00963 silencing lentivirus (SH-LINC00963), NOP2 silencing lentivirus (SH- NOP2), and the corresponding control lentivirus (NC) were obtained from GeneChem (Shanghai, China). Cells were seeded in 6-well plates (5 × 105 cells/well) before transduction. The shRNA transduction was conducted using the HiPerFect Transfection Reagent according to the manufacturer's instructions (Qiagen). Transduction with the lentiviral vectors was conducted using transduction reagents and 8 mg/ml polybrene (GeneChem) for 12 h. For viral transduction, cells were transduced with a multiplicity of infection (MOI) of 10,100 or 1,000. Overexpression, silencing, and the corresponding control stable cell lines were then established, and the efficiency of transduction was confirmed by RT-qPCR.

### Cell migration and cell invasion assays

Transwell migration and invasion assays were performed as previously described. DU 145 were seeded in the upper chambers, and the conditioned medium was placed in the lower chambers. Cells were stained with crystal violet and observed under an optical microscope. All experiments were performed in triplicate.

### Western blot assay and antibodies

DU 145 cells were lysed with RIPA extraction reagent (Beyotime) supplemented with a protease inhibitor cocktail (Roche). Cell protein lysates were separated by 10% sodium dodecyl sulfate-polyacrylamide gel electrophoresis (SDS-PAGE), transferred to 0.22 mm polyvinylidene fluoride membranes (Millipore), and probed with specific antibodies. Specific bands were detected by ECL chromogenic substrate and quantified by densitometry (Quantity One software, Bio-Rad). GAPDH antibody was used as the control. Antibodies against NOP2, GAPDH, E-cadherin, N-cadherin, vimentin, and β-actin were purchased from Cell Signaling Technology.

### RNA immunoprecipitation

RNA immunoprecipitation was used to investigate whether LINC00963 could interact or bind with the potential binding protein argonaute 2 (Ago2) in DU 145 and PC-3 cells. We used the EZMagna RIP Kit (Millipore) according to the manufacturer's instructions. DU 145 and PC-3 cells were lysed and incubated with protein A magnetic beads, which were conjugated with antibodies at 4 °C. After 3 to 6 hours, the beads were washed with wash buffer and then incubated with 0.1% SDS/0.5 mg mL-1 proteinase K for 30 minutes at 55 °C to remove proteins. Finally, immunoprecipitated RNA was subjected to RT-qPCR analysis to demonstrate the presence of LINC00963.

### Luciferase assay

The complementary DNA fragment containing the wild-type or mutant LINC00963 fragment and the 3’-untranslated region (UTR) of *NOP2* was subcloned downstream of the luciferase gene within the pGL3-Basic luciferase reporter vector (Promega). Human 293T cells (1.0*105) grown in a 24-well plate were co-transfected with 150 ng of either empty vector or miR-542-3p, 50 ng of firefly luciferase reporter comprising wild-type or mutant LINC01234 fragment, and the 3’-UTR of NOP2 fragment using Lipofectamine 3000 (Invitrogen). Forty-eight hours after transfection, luciferase assay was determined using the dual luciferase kit (Promega). The relative firefly luciferase activities were normalized to those of Renilla luciferase. Transfection was performed in triplicate.

### RNA sequencing

The RNA quality and integrity were analyzed by Qubit 2.0 (Life Technologies) and Bioanalyzer 2100 (Agilent). For library preparation, 3 μg total RNA was captured by NEBNext Oligo d (T) 25 beads (NEB), sheared to yield fragments of approximately 250 bp, and reverse transcribed using NEBNext RNA first and second Strand Synthesis Module (NEB, USA). The products were end-repaired, A-tailed, ligated to Illumina sequencing adapters and amplified by PCR. The quality of the sequencing library was assayed by using the Qubit 2.0 fluorometer (Life Technologies, USA) and the Bioanalyzer 2100 (Agilent) and then sequenced using an Illumina Hiseq X Ten with 2 × 150 bp paired-end sequencing, controlled by HiSeq Control Software (HCS). Raw sequence reads were initially examined using FastQC for quality control. Raw reads were processed to trim low-quality sequences and adapters using Trimmomatic. Clean reads were then mapped to hg19 for human samples and mm9 for mouse samples using STAR, and only uniquely mapped reads were kept. Read counts were calculated by htseq-count. Differential expression analysis was performed using DESeq2.

### Statistical analysis

The significance of differences between groups was assessed by a paired, two-tailed Student *t*-test. The univariate and multivariate Cox proportional hazards model was used to determine the effects of variables on survival. The Kaplan-Meier method test was utilized for survival analysis. Spearman correlation analysis was used to calculate the correlation between LINC00963, miR-542-3p, and NOP2. All statistical analyses were performed using SPSS 17.0 software. A P value of < 0.05 determined statistical significance.
